# IdentifyingInfluential Nodes in Complex Networks Based on the Integration of Smallest-Cycle and Non-Smallest-Cycle Features

**DOI:** 10.3390/e28080880

**Published:** 2026-08-05

**Authors:** Fu Tan, Xiaolong Chen, Ruijie Wang, Chi Huang, Shimin Cai

**Affiliations:** 1 School of Business Administration, Southwestern University of Finance and Economics, Chengdu 611130, China; ftan5967@gmail.com; 2 School of Computing and Artificial Intelligence, Southwestern University of Finance and Economics, Chengdu 611130, China; chenxiaolong@swufe.edu.cn (X.C.); huangchi@swufe.edu.cn (C.H.); 3 School of Mathematics, Aba Teachers College, Aba 623002, China; 4 Big Data Research Center, University of Electronic Science and Technology of China, Chengdu 611731, China; shimin.cai81@gmail.com

**Keywords:** complex network, influential node, smallest-cycle structures, non-smallest-cycle structures, weighted integration strategy

## Abstract

In complex network analysis, the identification of influential nodes is a fundamental issue, which is closely related to the structural robustness of the network and the dynamics of propagation processes. Current research primarily focuses on mesoscale features based on the smallest cycles or local features derived from star-shaped structures. However, the role of neighboring nodes that are connected to a given node but do not participate in its smallest cycles remains underexplored in network analysis. To address this issue, this paper proposes a hybrid centrality measure that integrates information from both smallest-cycle structures and non-smallest-cycle structures associated with each target node. The smallest-cycle structures considered in this method are identified only within the imposed local search range and do not necessarily correspond to the true smallest cycles in the full graph. Specifically, the extent of a node’s involvement in mesoscale structures is characterized by the number of the smallest cycles it participates in, while its local structural heterogeneity is represented by the number of neighboring nodes connected to it that do not belong to any smallest cycles. These two aspects are then unified into a single node importance metric through a weighted integration strategy. This paper evaluates node importance from multiple perspectives, including propagation capability analysis based on the SI model, network robustness testing through node attack simulations, and ranking accuracy assessment using Kendall correlation coefficient. The experimental results demonstrate that the proposed method achieves competitive or superior performance compared with the selected baseline methods under the experimental settings considered in this work. The findings indicate that integrating smallest-cycle and non-smallest-cycle features provides a more comprehensive characterization of a node’s role in complex networks. This study offers a novel perspective on the integration of multi-scale structural information in complex networks and presents an effective new approach for the identification of important nodes.

## 1. Introduction

Complex networks, as a fundamental framework for characterizing complex systems in the real world [1], have been extensively applied across various domains, including biological networks [2], transportation systems [3], social networks [4], and financial markets [5]. In these systems, the identification of node importance plays a critical role in understanding network structure [6], enhancing system robustness [7], and optimizing information dissemination [8]. For instance, in the process of epidemic spreading, identifying critical transmission nodes facilitates the development of effective intervention strategies, thereby reducing the scope and speed of the spread [9,10]. In infrastructure networks, locating key nodes contributes to enhancing the system’s resilience and fault tolerance, which prevents cascading failures [11]. In percolation theory, the identification of critical nodes plays an important role in analyzing the evolution of network connectivity, percolation phase transitions, and network robustness, thereby providing an important theoretical foundation for the structural optimization and functional maintenance of complex networks [12,13,14,15,16]. Therefore, accurately identifying influential nodes from the complex network is not only essential for a deeper understanding of the network topology but also directly impacts the ability to control dynamic processes. This topic remains a central issue in complex network research and continues to attract significant attention.

In recent years, various methods for measuring node importance have been proposed from different perspectives. Traditional approaches have been predominantly founded on local or global topological properties. For instance, degree centrality [17] is defined by the number of a node’s immediate connections, which represents the local influence. The community-structure-based centrality [18,19] identifies key nodes within local communities by analyzing the structural positions of nodes in communities. Betweenness centrality [20] is quantified by the extent to which a node functions as an intermediary along the shortest paths, which reflects the node’s capacity to regulate information flow. Closeness centrality [21] is assessed based on the average shortest distance between a node and all other nodes; it indicates its overall proximity within the network. Subsequently, methods such as the coreness metric based on K-shell decomposition [22] and the H-index [23] have been introduced to further characterize node importance from the perspectives of network hierarchical structure and neighborhood features. Recognizing that a single topological feature is often insufficient to comprehensively characterize the structural role of nodes in complex networks, researchers have proposed a variety of hybrid centrality measures that integrate complementary topological features to achieve more accurate and robust evaluations of node importance [24,25,26]. These methods have, to some extent, improved computational efficiency and identification performance. However, they fundamentally rely on the assumption of a “star structure” or direct neighborhood influence, where a node’s impact is considered to stem primarily from its immediate connections. However, these methods often overlook more complex mesoscale structural information in networks, particularly the closed interactive relationships among multiple nodes. As a result, they exhibit certain limitations in real-world networks characterized by strong local clustering or redundant connectivity patterns [27,28].

To address this limitation, recent studies have gradually shifted attention from traditional chain-like and star-like structures to cycle structures [29,30]. A cycle is defined as a closed path in which the starting and ending nodes overlap, while all intermediate nodes are non-repeating. Its simplest form is a triangle. Unlike chain structures that support only a single information propagation pathway, the cycle structures provide multiple independent paths between nodes, which have the capability of path redundancy and feedback mechanisms. These features play a crucial role in maintaining network connectivity and shaping dynamical evolution processes on networks [31]. For instance, when two nodes belong to the same cycle, even in the absence of a direct edge between them, indirect interactions can still be established through multiple alternative paths. Such “indirect interactions” significantly enhance the overall coupling strength and structural cohesion of the network topology. Fan et al. [32] further extended this perspective by introducing cycle-based metrics, such as the smallest cycles and cycle ratio, to quantify the extent to which nodes participate in fundamental cycle structures. The effectiveness of these measures was validated in node attack and synchronization control experiments, where they demonstrated superior performance. In addition, related studies have demonstrated that cycle structures play a crucial role in network synchronization [33], information storage capacity [34], and structural stability [35]. As a representative mesoscale structure, cycles can effectively bridge the cognitive gap between traditional local and global metrics. Nevertheless, existing cycle-based methods mainly focus on quantifying the extent to which nodes participate in the smallest cycles [36,37,38], while paying insufficient attention to neighboring nodes that are connected to a given node but not involved in the smallest cycles. Consequently, an important class of structural information is largely overlooked.

In fact, in complex networks, in addition to the smallest cycle structures, there also widely exist a large number of neighboring nodes that do not participate in the smallest cycles. These nodes typically form tree-like or chain-like branching structures and are extensively distributed in the peripheral or transitional regions of the network [39]. Although these nodes do not participate in the smallest cycles, they still play a non-negligible role in information propagation and structural support [40]. For example, in spreading dynamics, these non-smallest-cycle nodes often serve as frontier channels in the diffusion process [41], determining the coverage range of the propagation; structurally, they connect core regions with peripheral regions, maintaining the overall connectivity and hierarchical organization of the network [42,43]. Furthermore, in certain sparse or tree-like networks, non-smallest-cycle structures can even dominate [44]. However, existing research mostly treats these non-smallest-cycle structures as secondary factors, lacking systematic characterization of their structural roles and effective methods for unified modeling and analysis together with the smallest cycle structures. This deficiency, to some extent, limits more comprehensive and accurate assessments of node importance, leaving room for improvement in the performance of existing methods on networks with complex structures.

To address the above issues, this paper proposes a novel node importance measure that integrates information from both smallest-cycle and non-smallest-cycle structures. The smallest-cycle structures considered in this method are identified only within the imposed local search range and do not necessarily correspond to the true smallest cycles in the full graph. Specifically, the Number of Smallest Cycles involving a node is first employed to characterize its role in mesoscale closed structures, thereby reflecting its participation in multi-path interactions and feedback mechanisms. Subsequently, the number of neighboring nodes that are directly connected to the target node but do not belong to any of its smallest-cycle structures is calculated to characterize the node’s non-smallest-cycle structural feature, which complements the structural information overlooked by conventional cycle-based measures. Finally, these two complementary structural features are integrated through a weighted fusion strategy to construct a unified node importance metric, enabling a comprehensive utilization of structural information across multiple scales. Compared with existing methods, the main contributions of this work are summarized as follows:

(1) A unified analytical framework is proposed to jointly characterize the smallest-cycle structures associated with a target node and their complementary non-smallest-cycle neighboring structures. Unlike existing cycle-based methods that mainly focus on the smallest-cycle structures, the proposed framework enables a more comprehensive representation of node importance by integrating complementary structural information from local neighborhoods to mesoscale structures;

(2) The number of non-smallest-cycle neighboring nodes is introduced as a complementary structural descriptor to capture structural information that is overlooked by conventional cycle-based measures. This complementary feature effectively enhances the discriminative capability of node importance evaluation, particularly in networks where non-smallest-cycle structures are prevalent;

(3) Extensive experiments on six real-world networks demonstrate that the proposed method achieves competitive or superior performance compared with the selected baseline methods under the experimental settings considered in this work, from the perspectives of both structural robustness and spreading dynamics, thereby verifying the effectiveness and general applicability of the proposed framework.

To validate the performance of the proposed method, extensive experiments are conducted on six real-world networks, and node importance is evaluated from multiple perspectives. Specifically, a diffusion capability analysis based on the SI model is performed to measure the influence scope and spreading speed of nodes during the propagation process [45]. A network connectivity assessment under targeted attacks is further carried out, where variations in the size of the largest connected component during node removal are analyzed to characterize the impact of nodes on structural robustness [46]. In addition, a rank correlation analysis based on Kendall’s coefficient is employed to evaluate the consistency among different metrics and their agreement with real diffusion dynamics [47]. The experimental results demonstrate that the proposed method achieves competitive or superior performance compared with the selected baseline methods across different network types and evaluation criteria under the experimental settings considered in this work. These results further demonstrate that integrating the structural features of smallest-cycle and non-smallest-cycle structures enables a more comprehensive characterization of the roles of nodes in complex networks and provides a more effective analytical framework for influential node identification.

The remainder of this paper is organized as follows. Section 1 introduces the fundamental definitions of complex networks and cycle structures, and presents the theoretical foundations for node importance analysis. Section 2 elaborates on the proposed method in detail, including the characterization of the smallest cycle numbers of nodes, the definition of non-smallest-cycle neighbor features, and the construction of the weighted fusion strategy. Section 3 describes the experimental design and evaluation metrics, which contains diffusion capability analysis based on the SI model, network connectivity assessment under targeted attacks (measured by the size of the largest connected component), and rank consistency analysis based on Kendall’s coefficient. Section 4 presents the experimental results on six real-world networks and compares the proposed method with various classical approaches as well as existing cycle-based metrics. Finally, Section 5 concludes the paper and discusses potential directions for future research.

## 2. Preliminaries

The complex network can be formally represented as an undirected and unweighted graph G=(V,E), where $V=\left\{v_{1},v_{2},\ldots ,v_{N}\right\}$ denotes the set of nodes, and E⊆V×V represents the set of edges. Here, N=|V| denotes the total number of nodes in the network. If there exists a connection between nodes $v_{i}$ and $v_{j}$, it is denoted as $\left(v_{i},v_{j}\right)\in E$. On this basis, the concept of a cycle is introduced to describe closed structures within the network. Specifically, in graph *G*, if there exists a path composed of a sequence of nodes—$C:v_{i_{1}}\to v_{i_{2}}\to \ldots \to v_{i_{k}}\to v_{i_{1}}$—and all nodes along the path are distinct except that the starting and ending nodes coincide, the path is defined as a cycle. The size of a cycle is defined as the number of edges it contains (or equivalently, the number of nodes), i.e., |C|=k. As a representative mesoscopic structure in complex networks, cycle structures capture indirect relationships among nodes and reflect the redundancy and stability of local topological configurations.

### 2.1. Centrality Measures

To comprehensively evaluate the effectiveness and robustness of the proposed method, several representative node centrality measures are selected for comparative analysis. These measures characterize the structural importance of nodes in complex networks from different perspectives. Specifically, the comparison methods including the Semi-local Centrality (SC) [48], Weighted Shortest Path Length Centrality (WSLC) [49], K-shell decomposition (KS) [22], Fusion Gravity Model (FGM) [50], Eigenvector Centrality (EC) [51], and the Number of Smallest Cycles (NC) [32]. These metrics are widely used in complex network analysis and are grounded in diverse theoretical foundations, which provide complementary structural insights.

#### 2.1.1. The Semi-Local Centrality (SC)

Semi-local Centrality (SC) [48] is an extended centrality measure designed to quantify the local influence of a node. Compared with degree centrality, which relies solely on the number of direct neighbors, SC incorporates second-order neighborhood information, thereby providing a more comprehensive characterization of node importance. Specifically, this metric is defined by aggregating the degree values of a node’s neighbors as well as those of their adjacent nodes, and it can be expressed as follows:

(1) $$SC\left(v_{i}\right)=\sum_{v_{j}\in \Gamma \left(v_{i}\right)}\sum_{v_{\ell }\in \Gamma \left(v_{j}\right)}k\left(v_{\ell }\right),$$

where $\Gamma \left(v_{j}\right)$ denotes the set of first-order neighbors of node $v_{i}$, and $k\left(v_{l}\right)$ represents the degree of node $v_{l}$. This definition indicates that the importance of a node depends not only on the number of its direct neighbors but is also influenced by the connectivity scale of its second-order neighbors. Consequently, it enables a more effective characterization of a node’s diffusion capability and structural role within the local network.

#### 2.1.2. The Weighted Shortest Path Length Centrality (WSLC)

The Weighted Shortest Path Length Centrality (WSLC) [49] is proposed to integrate local structural information, semi-local topological features, and global sensitivity based on the average shortest path, which provides a comprehensive characterization of node importance. It is defined as follows:

(2) $$\mathrm{WSLC}\left(\left(v_{i}\right)\right)=\alpha_{1}Q_{\mathrm{Local}}\left(i\right)+\alpha_{2}Q_{\mathrm{Semi}\text{-}\mathrm{Local}}\left(i\right)+\alpha_{3}Q_{\mathrm{LRASP}},$$

where $Q_{\mathrm{Local}}$ denotes the local influence term, $Q_{\mathrm{Semi}\text{-}\mathrm{Local}}$ represents the semi-local influence term, and $Q_{\mathrm{LRASP}}\left(i\right)$ corresponds to the structural sensitivity term. The parameters $\alpha_{1}$, $\alpha_{2}$, and $\alpha_{3}$ are weighting coefficients, with $\alpha_{1}=\alpha_{2}=\alpha_{3}=1/3$.

#### 2.1.3. The K-Shell Decomposition (KS)

The K-shell Decomposition (KS) [22] is employed to quantify the core hierarchical position of nodes in complex networks. By recursively removing low-degree nodes, the network is decomposed into multiple shell layers, thereby assigning a shell index to each node. The core definition is given as follows:

(3) $$KS\left(v_{i}\right)=ks\left(i\right),$$

where KS(i) denotes the K-shell value of node *i*, and ks(i) represents the shell index to which the node belongs. A larger value indicates that the node is located closer to the core of the network and plays a more significant role in structural stability and information diffusion within the network.

#### 2.1.4. The Fusion Gravity Model (FGM)

Motivated by the law of universal gravitation, node influence is characterized via a mass–distance interaction mechanism. Let $q_{i}$ and $q_{j}$ denote the fusion (mass) values associated with nodes $v_{i}$ and $v_{j}$, respectively, while $d_{ij}$ represents the shortest path distance between them. The interaction range is controlled by a cutoff radius *R*, and $\lambda_{i}$ is a normalized weighting coefficient assigned to node $v_{i}$. Based on this formulation, the gravitational influence of node $v_{i}$ is defined as follows [50]:

(4) $$\mathrm{FGM}\left(v_{i}\right)=\lambda_{i}\sum_{\begin{array}{c} j\neq i\\ d_{ij}\leq R \end{array}}\frac{q_{i}\cdot q_{j}}{d_{ij}^{2}}.$$

The fusion value $q_{i}$ of node $v_{i}$ is constructed as follows:

(5) $$q_{i}=\frac{k_{i}}{k_{\max}}+\frac{ks_{i}}{ks_{\max}},$$

where $k_{i}$ denotes the degree of node $v_{i}$, $k_{\max}$ is the maximum node degree in the network, $ks_{i}$ refers to the *K*-shell index of node $v_{i}$, and $ks_{\max}$ is the maximum *K*-shell value in the system. All variables are normalized to eliminate scale effects and ensure comparability across metrics.

#### 2.1.5. The Eigenvector Centrality (EC)

Eigenvector Centrality [51] is based on the principle that “important nodes tend to be connected to other important nodes,” and it evaluates node importance by accounting for the influence of its neighbors. Let *A* denote the adjacency matrix of the network, *x* be the eigenvector, and λ represent the largest eigenvalue. The Eigenvector Centrality score of node $v_{i}$ is defined as follows:

(6) $$EC\left(v_{i}\right)=x_{i},$$

where the eigenvector *x* satisfies the eigenvalue equation Ax=λx. In this expression, $x_{i}$ denotes the component of the eigenvector corresponding to node $v_{i}$. A larger value of $x_{i}$ indicates a higher level of node influence. Eigenvector Centrality takes into account not only the direct neighbors of a node but also the influence of indirect neighbors, and compared with degree centrality, it provides a more comprehensive characterization of the global importance of nodes in the network.

#### 2.1.6. The Number of Smallest Cycles (NC)

For any node $v_{i}\in V$, let its associated cycle set be defined as $C_{i}=\left\{C|v_{i}\in C\right\}$, which includes all closed-loop structures in which node $v_{i}$ participates within the network. Based on this, the concept of the smallest cycle is introduced to characterize the most compact closed structure embedded around a node. Specifically, the smallest cycle length of node $v_{i}$ is defined as follows:

(7) $$g_{i}=\min_{C\in C_{i}}\left|C\right|,$$

That is, the smallest cycle length of node $v_{i}$ corresponds to the smallest size among all cycles in which node $v_{i}$ participates. Furthermore, the smallest cycle set of node $v_{i}$ can be defined as $S_{i}=\{C\in C_{i}|\left|C\right|=g_{i}\}$, which contains all cycles that include node $v_{i}$ and attain the smallest cycle size. Based on this, the Number of Smallest Cycles associated with node $v_{i}$ is defined as the cardinality of this set [32]:

(8) $$NC\left(v_{i}\right)=\left|S_{i}\right|.$$

It is worth noting that, when node $v_{i}$ does not belong to any cycle, $C_{i}=\emptyset$, the node is located in a tree-like or bridge structure and does not participate in any closed paths. In this case, no smallest cycle exists for the node. Consequently, we have $S_{i}=\emptyset$ and $NC(v_{i})=0$.

## 3. The Proposed Method

### 3.1. Integrate of Smallest-Cycle and Non-Smallest-Cycle Features (CNC)

To systematically characterize node importance in complex networks, this paper proposes a hybrid centrality measure that integrates information from both smallest-cycle structures and non-smallest-cycle structures associated with each target node. The smallest-cycle structures considered in this method are identified only within the imposed local search range and do not necessarily correspond to the true smallest cycles in the full graph. The core idea is as follows. In complex network analysis, the identification of critical nodes is closely related to structural robustness and spreading dynamics. Existing studies have primarily focused on mesoscopic features based on the smallest cycle structures or local connectivity patterns derived from star-like structures, while the potential role of neighboring nodes that are connected to the target node but do not participate in its smallest cycles has largely been overlooked. To address this issue, this study incorporates both smallest cycles participation and non-smallest cycles neighbor features to provide a more comprehensive characterization of the structural roles of nodes.

Specifically, the computation procedure of the CNC method can be summarized as follows. First, a fixed search step is assigned to each node (set to 8 in this study) to constrain the scope of local structural analysis. Second, under this step-size constraint, the Number of Smallest Cycles involving the target node is counted to characterize its embedding level within mesoscopic closed structures. Third, the number of neighboring nodes directly connected to the target node but not participating in any smallest cycles is calculated, which reflects the heterogeneity of its local structural configuration and its boundary-expansion capability. Finally, these two types of structural information are integrated through a weighted fusion strategy to construct a unified metric for node importance.

**Step 1: Set a fixed search step**

The proposed method is designed to characterize the local structural organization surrounding each target node rather than to exhaustively enumerate all cycle structures in the entire network. In principle, identifying the global smallest cycles associated with every node would require searching paths between the target node and a large number of other nodes across the whole network. Such exhaustive exploration leads to a substantial computational burden, especially for large-scale sparse networks, making the algorithm computationally expensive and difficult to scale.

Moreover, although two distant nodes may theoretically belong to the same smallest-cycle structure, their mutual structural influence is generally very weak due to the long shortest path distance separating them. Since influential node identification primarily depends on local and mesoscale structural interactions, considering extremely long-range cycle structures usually provides limited additional information while significantly increasing the computational cost.

A fixed search step of 8 is assigned to each node to constrain the scope of local structural analysis. This setting is based on the six degrees of separation principle [52], which states that, on average, information between any two nodes in a complex network passes through approximately six intermediate nodes. By including both the source and target nodes, the effective path length is about 8. Within this range, nodes still exert significant influence on each other, whereas influence beyond this length can be considered negligible. Thus, setting the search step to 8 not only aligns with the fundamental characteristics of small-world networks but also effectively reduces the search scope; it can lower the computational complexity.

**Step 2: Compute the Number of Smallest Cycles**

Within a local search radius defined by a step size of 8, cycle detection is first performed for each node. During this process, once a smallest cycle containing the target node is identified within the reachable range (i.e., the shortest closed path within the current search scope), the search is not extended to larger scales. Instead, the size of this smallest cycle is fixed, and the number of occurrences of such smallest cycles involving the node is counted to quantify its participation intensity within compact closed structures. If no cycle structure is detected within the step size of 8, the node is considered not to participate in any local closed structure, and its smallest cycle count is defined as 0. Based on the above definition, the Number of Smallest Cycles of the node is expressed as follows:

(9) $$NC\left(v_{i}\right)=\left|S_{i}\right|,$$

This metric is used to characterize the embedding level and repeated participation of a node within the most compact closed structures under a locally constrained range.

**Step 3: Computate the non-smallest-cycle neighbor nodes**

In this step, the first-order neighbors of the target node $v_{i}$ are identified. Subsequently, among these neighbors, nodes that do not participate in any smallest-cycle structure centered on $v_{i}$ are selected, and their number is counted. These nodes represent connections around the target node that are not covered by compact closed-loop structures, which capture the heterogeneity of its local topology as well as its potential for outward expansion. Specifically, this measure is obtained by excluding nodes involved in smallest-cycle structures from the neighbor set, and it can be expressed as follows:

(10) $$NN\left(v_{i}\right)=\left|\Gamma \left(v_{i}\right)\setminus V\left(S_{i}\right)\right|,$$

where $\Gamma \left(v_{i}\right)$ denotes the set of first-order neighbors of node $v_{i}$, and $V\left(S_{i}\right)$ represents the set of all nodes involved in smallest-cycle structures. This metric is used to characterize the scale and structural heterogeneity of the node’s neighborhood that is not constrained by closed-cycle structures in its local connectivity pattern.

**Step 4: Integrate the smallest-cycle structures and the non-smallest-cycle structures**

In complex networks, the importance of a node is not only determined by its participation in smallest-cycle structures but also by its role in non-smallest-cycle structures. Specifically, the smallest-cycle structures capture the embedding capability of a node within local fundamental closed-loop configurations, which reflect its core role in maintaining structural constraints at the local level. In contrast, the non-smallest-cycle structures describe the node’s contribution to the larger-scale redundant paths and extended connectivity, which reflects its supplementary role in global structural expansion and multi-path accessibility.

Specifically, the proposed CNC index is designed to integrate two complementary types of structural information associated with a target node. The smallest-cycle structures characterize the extent to which a node participates in densely interconnected local regions, reflecting its structural embeddedness and local reinforcement capability. In contrast, the non-smallest-cycle neighboring structures describe the complementary branching structures surrounding the target node, providing additional information on potential spreading pathways and structural connectivity beyond the smallest-cycle structures. Since these two structural descriptors capture different yet complementary aspects of node importance, their weighted linear combination enables both types of structural information to contribute simultaneously to the final importance evaluation while maintaining good physical interpretability and computational simplicity. Compared with nonlinear fusion or more sophisticated integration strategies, the weighted linear combination preserves the individual contributions of the two structural descriptors, avoids introducing unnecessary model complexity, and facilitates parameter interpretation and sensitivity analysis. Therefore, for influential node identification in complex networks, the weighted linear integration provides a reasonable and practical strategy for combining complementary structural information.

**Figure 1:**
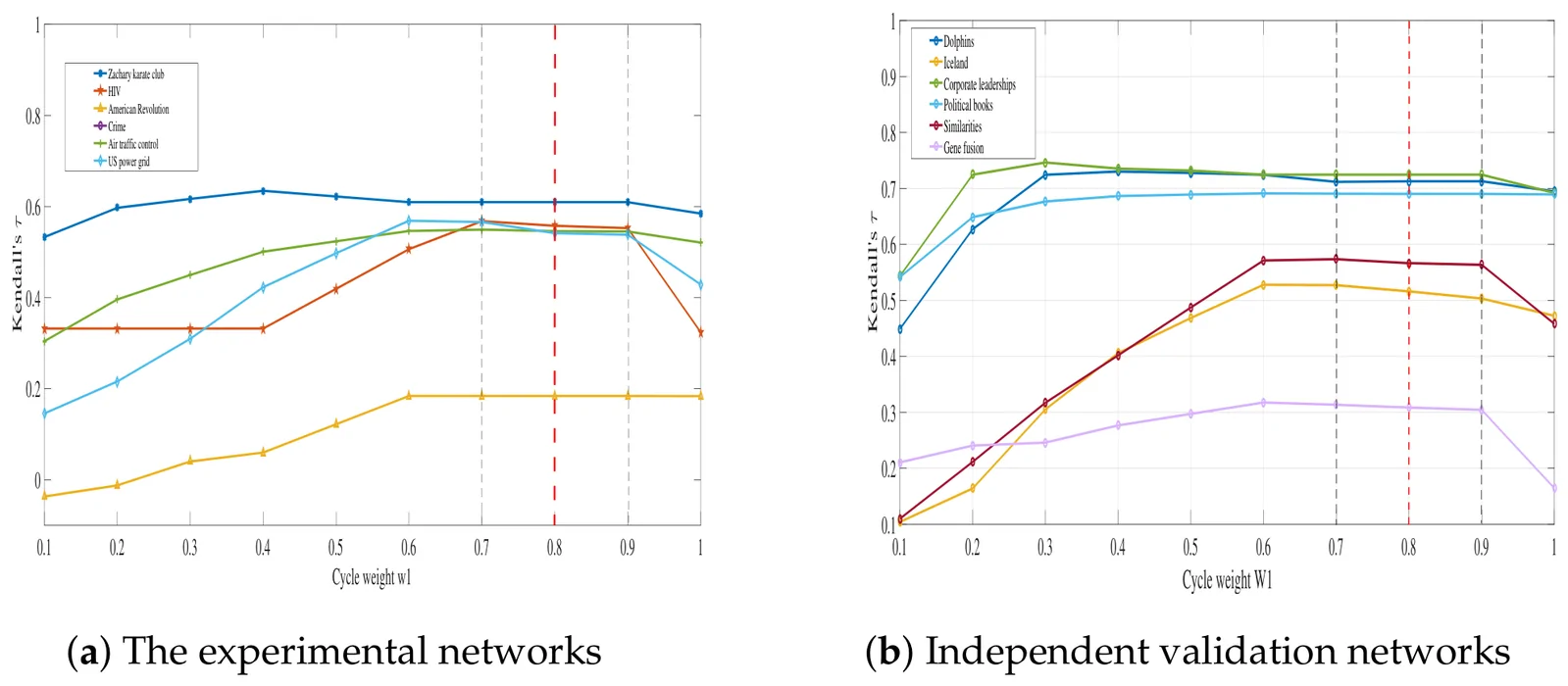
Sensitivity analysis of the proposed method with respect to the cycle weight $w_{1}$. (**a**) Experimental networks; (**b**) independent validation networks. The Kendall correlation coefficient τ between the node rankings generated by the proposed method and those obtained from the SI model is plotted as a function of $w_{1}$ for six additional real-world networks.

To comprehensively characterize a node’s role across these two structural types, a weighted fusion strategy is adopted. The contribution of smallest-cycle structures $NC\left(v_{i}\right)$ and that of non-smallest-cycle structures $NN\left(v_{i}\right)$ are linearly combined. A weight of $w_{1}$ is assigned to $NC\left(v_{i}\right)$, while a weight of $w_{2}$ is assigned to $NN\left(v_{i}\right)$, emphasizing the dominant role of smallest-cycle structures in local core organization. The formulation is given as follows:

(11) $$CNC\left(v_{i}\right)=w_{1}NC\left(v_{i}\right)+w_{2}NN\left(v_{i}\right).$$

where $CNC\left(v_{i}\right)$ denotes the comprehensive structural centrality of node $v_{i}$, $w_{1}+w_{2}=1$. This weighting scheme effectively integrates both the core role in smallest-cycle structures and the complementary role in non-smallest-cycle structures, which improves the expressive power and discriminative capability of node importance evaluation. To determine the optimal weight parameters, Kendall’s tau correlation analysis is conducted between the node rankings obtained under different weight combinations and the influence rankings generated by the SI model. As shown in the Figure 1a, the Kendall’s tau coefficient continuously increases as the cycle-structure weight $w_{1}$ increases from 0.1 to 0.7, indicating that cycle-structure information increasingly enhances the ability to characterize node influence and improves the consistency between the obtained rankings and the spreading results of the SI model. When $w_{1}$ lies within the range of 0.7 to 0.9, the Kendall’s tau coefficient remains nearly unchanged and reaches its maximum value, suggesting that the proposed method achieves both optimal and stable performance in this interval. However, when $w_{1}$ exceeds 0.9, the Kendall’s tau coefficient gradually decreases, indicating that overemphasizing cycle-structure features weakens the contribution of non-cycle structural information and consequently reduces the correlation between the ranking results and the actual spreading capability of nodes. Considering both ranking performance and parameter stability, the midpoint of the optimal stable interval [0.7,0.9] is selected as the cycle-structure weight, i.e., $w_{1}=0.8$, while the non-cycle-structure weight is set to $w_{2}=0.2$. The experimental results show that the node rankings obtained with $w_{1}=0.8$ and $w_{2}=0.2$ achieve the highest and most stable consistency with the SI-based rankings. Therefore, this parameter combination is adopted in all subsequent experiments.

Specifically, we select Dolphins, Iceland, Corporate leaderships, Political books, Similarities, and Gene fusion networks to determine the weight parameters through Kendall correlation analysis with the SI model. As shown in Figure 1b, the results demonstrate that the obtained weight settings remain consistent with those derived from the original experiments, confirming the robustness and rationality of the proposed parameter selection strategy.

### 3.2. Computational Complexity Analysis

In terms of computational complexity, the proposed CNC method maintains high computational efficiency while providing a more comprehensive structural characterization than the original NC method. The dominant computational cost of CNC arises from the BFS-based smallest-cycle search, whose time complexity is $O(Nk^{L-1})$, where *k* denotes the average node degree and the maximum search depth is fixed at L=8. Compared with NC, CNC introduces only two additional operations, namely non-smallest-cycle neighboring structure statistics and weighted feature fusion, with time complexities of O(M) and O(N), respectively. Since these additional operations are local computations, they do not alter the asymptotic time complexity of the algorithm. Therefore, CNC retains the same computational order as NC. In contrast, the SC method repeatedly computes shortest paths between each node and all other nodes, resulting in an overall computational complexity of approximately $O\;\left(N^{2}\left(M+N\log N\right)\right)$. Similarly, WSLC not only requires all-pairs shortest path computation but also repeatedly reconstructs local subgraphs and recalculates shortest paths during the LRASP computation, leading to the same overall complexity of approximately $O\;\left(N^{2}\left(M+N\log N\right)\right)$. FGM requires K-shell decomposition, Eigenvector Centrality computation, and all-pairs shortest path calculation, yielding a computational complexity of approximately $O\;\left(N\left(M+N\log N\right)+N^{2}\right)$. The computational cost of EC (Eigenvector Centrality) is dominated by the computation of the principal eigenvector of the adjacency matrix, resulting in a complexity of approximately $O(N^{3})$, which can be reduced to O(MI) using iterative methods, where *I* denotes the number of iterations. By comparison, K-shell decomposition performs only iterative node peeling, where each edge is processed at most once, resulting in a linear complexity of O(N+M). Overall, CNC preserves the same asymptotic computational complexity as NC while incorporating complementary non-smallest-cycle neighboring structural information through only a small amount of additional local computation. Compared with methods that rely on repeated global shortest path computations or eigenvalue decomposition, CNC achieves a favorable balance between computational efficiency and node importance characterization without increasing the asymptotic computational complexity.

### 3.3. Illustrative Example

In this paper, a hybrid node importance evaluation method, termed CNC, is proposed, which integrates information from the smallest-cycle structures and the non-smallest-cycle structures. The method is designed to simultaneously characterize the embedding role of nodes within the smallest-cycle structures and their extended connectivity in the non-smallest-cycle structures, thereby providing a more comprehensive representation of their structural roles in complex networks. To illustrate the computational procedure, the classical Zachary karate club network is employed as an example. Under a fixed search step of 8, the smallest-cycle structures associated with each node are first identified and their number $NC\left(v_{i}\right)$ is computed. Subsequently, the number of neighboring nodes that do not participate in any smallest-cycle structure, denoted as $NN\left(v_{i}\right)$, is determined. These two measures are then integrated through a weighted fusion scheme to obtain the composite indicator $CNC\left(v_{i}\right)$. This procedure enables effective discrimination of nodes that are both embedded in key smallest-cycle structures and possess certain structural expansion capability, thereby improving the completeness and accuracy of node importance evaluation.

**Figure 2:**
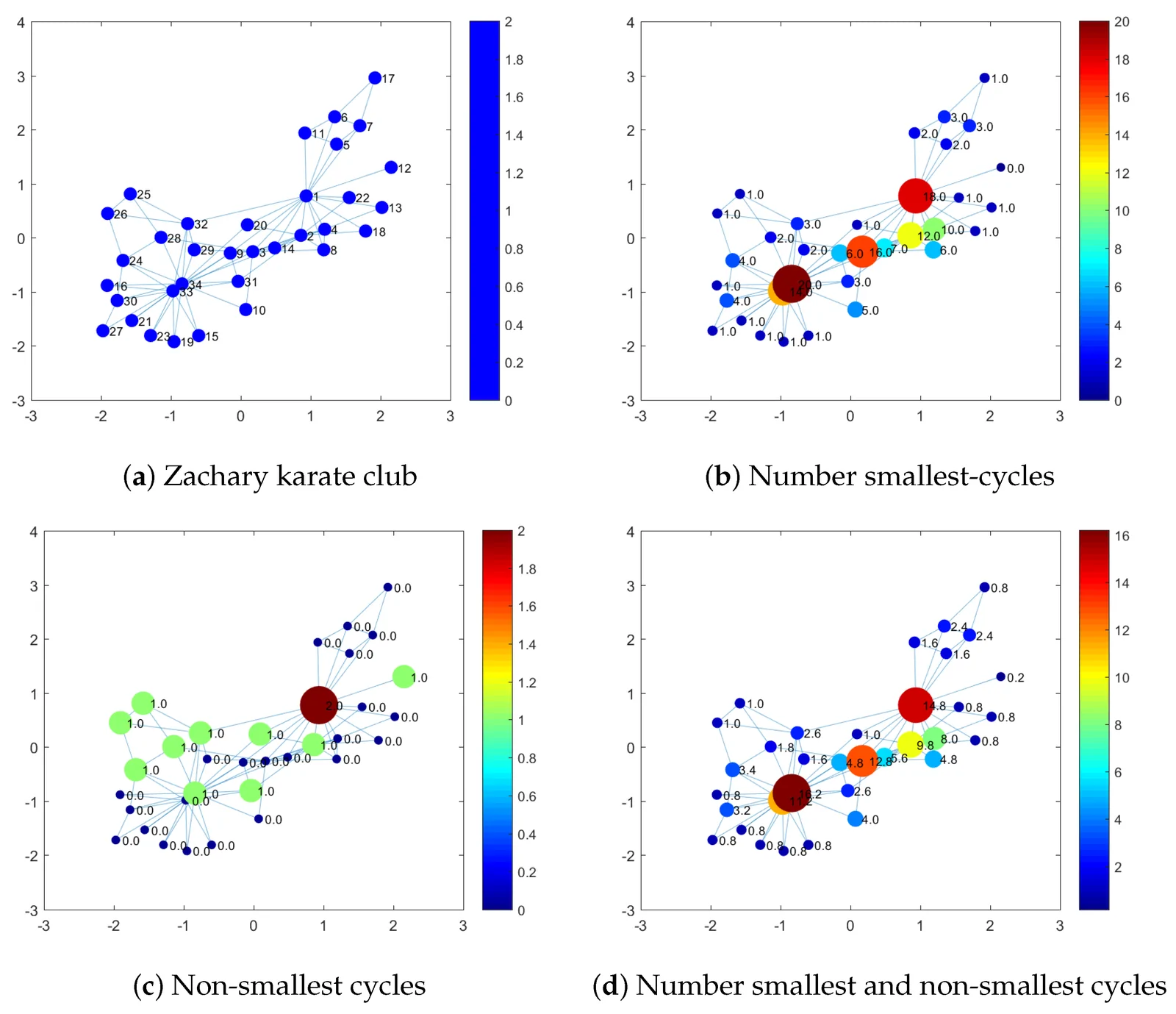
Visualization of the CNC method on the Zachary karate club network. (**a**) Original network with node indices; all nodes are colored blue. (**b**) Number of the smallest-cycle structures $NC\left(v_{i}\right)$. (**c**) Number of the non-smallest-cycle neighbor nodes $NN\left(v_{i}\right)$. (**d**) Composite indicator $CNC\left(v_{i}\right)$ after weighted fusion.

Figure 2 presents the computational results of the CNC method and their visual representations based on this network. Figure 2a shows the original network structure, where the numbers on the nodes denote node indices, and all nodes are uniformly colored in blue. Figure 2b illustrates the number of the smallest-cycle structures associated with each node, which is denoted as $NC\left(v_{i}\right)$. The color and size of nodes are positively correlated with their values, such that larger values correspond to a deeper red color and larger node size, that highlight their roles in smallest-cycle structures. Figure 2c displays the number of neighboring nodes that do not participate in any smallest-cycle structure, denoted as $NN\left(v_{i}\right)$. A similar visualization scheme is adopted, where both color and size increase with the values, which reflect the connectivity characteristics of nodes in the non-smallest-cycle structures. Figure 2d presents the composite indicator $CNC\left(v_{i}\right)$, which is obtained through weighted fusion, using the same visual encoding, thereby intuitively illustrating the overall structural importance of nodes in the network.

Taking node 6 as an example, it can be observed from Figure 2b that all the smallest cycles it participates in are triangular structures, with a corresponding value of $NC\left(v_{6}\right)=3$. Meanwhile, Figure 2c shows that none of its neighbors belong to non-smallest-cycle structures, i.e., $NN\left(v_{6}\right)=0$. In contrast, node 12 does not participate in any cycle in Figure 2b, resulting in $NC\left(v_{12}\right)=0$. If the evaluation were based solely on cycle structures, the importance of this node would be neglected, which is clearly inconsistent with its actual role in the network. However, as shown in Figure 2c, node 12 is still connected to neighbors that do not participate in smallest-cycle structures (e.g., node 1), yielding $NN\left(v_{12}\right)=1$, which indicates that it still plays a certain role in non-smallest-cycle structures. Based on this observation, the CNC metric is constructed by integrating these two types of structural information through a weighted scheme. As illustrated in Figure 2d, the composite value of node 6 is $CNC\left(v_{6}\right)=2.4$, while that of node 12 is $CNC\left(v_{12}\right)=0.2$. This formulation enables a unified characterization of different structural contributions and avoids the limitations associated with relying on a single structural feature.

This example not only facilitates understanding of the computational logic of the CNC method but also provides an intuitive reference for its application to larger-scale complex networks. Empirical results indicate that the proposed method exhibits strong capability in identifying key nodes across various complex systems, including social, transportation, and financial networks, and can effectively reveal the potential roles of nodes in structural stability and diffusion processes.

## 4. Experiment

We systematically evaluate the effectiveness and robustness of the proposed method. Specifically, three types of experiments are conducted to assess node importance. First, spreading simulations based on the Susceptible–Infected (SI) model are performed to analyze the influence of different methods in information diffusion processes. Second, network robustness experiments under node attack strategies are carried out, where the performance of each method is evaluated by examining the decline in the size of the largest connected component during the removal of critical nodes, thereby reflecting their capability to characterize structural vulnerability. Third, the Kendall coefficient is introduced to measure the consistency of ranking results, enabling the assessment of correlation and reliability among different centrality measures.

To comprehensively demonstrate the advantages of the proposed approach, six classical centrality measures are employed as comparative methods for horizontal evaluation. In addition, to ensure computational stability and reproducibility, all experiments are implemented on a recent version of MATLAB R2024a (MathWorks, Natick, MA, USA), with a computing environment consisting of an Intel Core i7 processor, 32 GB of RAM, and a 64-bit Windows 10 operating system. Based on this experimental framework, the applicability and superiority of the proposed method in complex networks are thoroughly validated through multi-perspective and multi-level analyses.

### 4.1. Datasets

Six real-world complex network datasets are selected, all of which are undirected and unweighted, covering several representative domains, including social relationships, biological systems, and infrastructure (http://konect.cc/networks/; accessed on 1 June 2026). The fundamental topological characteristics of these networks are summarized in Table 1, including the number of nodes *N*, the number of edges *E*, the average degree 〈k〉, the average shortest path length 〈d〉, the average clustering coefficient *C*, the network density ρ, and the network diameter *L*. These metrics collectively provide a multi-dimensional characterization of network structural properties.

Specifically, for small-scale networks, the Zachary karate club network consists of 34 nodes and 78 edges, with an average degree of 4.588, indicating relatively strong connectivity among nodes. Its clustering coefficient reaches 0.571, revealing a pronounced local clustering structure, which is typical of highly clustered small social networks. The HIV network comprises 40 nodes and 41 edges, with a low average degree of 2.05, suggesting sparse connectivity. Meanwhile, its average shortest path length is 4.473 and the network diameter is 10, reflecting relatively long information propagation paths and a loosely connected structure. The American Revolution network contains 136 nodes and 157 edges, with an average degree of 2.309 and a clustering coefficient of 0.093, indicating the presence of some local clustering while remaining globally sparse.

For medium- and large-scale networks, the Crime network includes 829 nodes and 1473 edges, with an average degree of 3.554 and an average shortest path length of 5.04. Its clustering coefficient is only 0.006, indicating extremely weak local clustering and a structure closer to that of a random network. The Air traffic control network consists of 1226 nodes and 2408 edges, with an average degree of 3.928, an average shortest path length of 5.929, and a network diameter of 17, maintaining relatively good connectivity and transmission efficiency despite its larger size. The US power grid network, as the largest dataset, contains 4941 nodes and 6594 edges, with an average degree of 2.669 and a very low network density of 0.001. Its average shortest path length reaches 18.989, and the network diameter is 46, exhibiting typical characteristics of a large-scale sparse network, including long information transmission paths and a highly dispersed structure.

**Figure 3:**
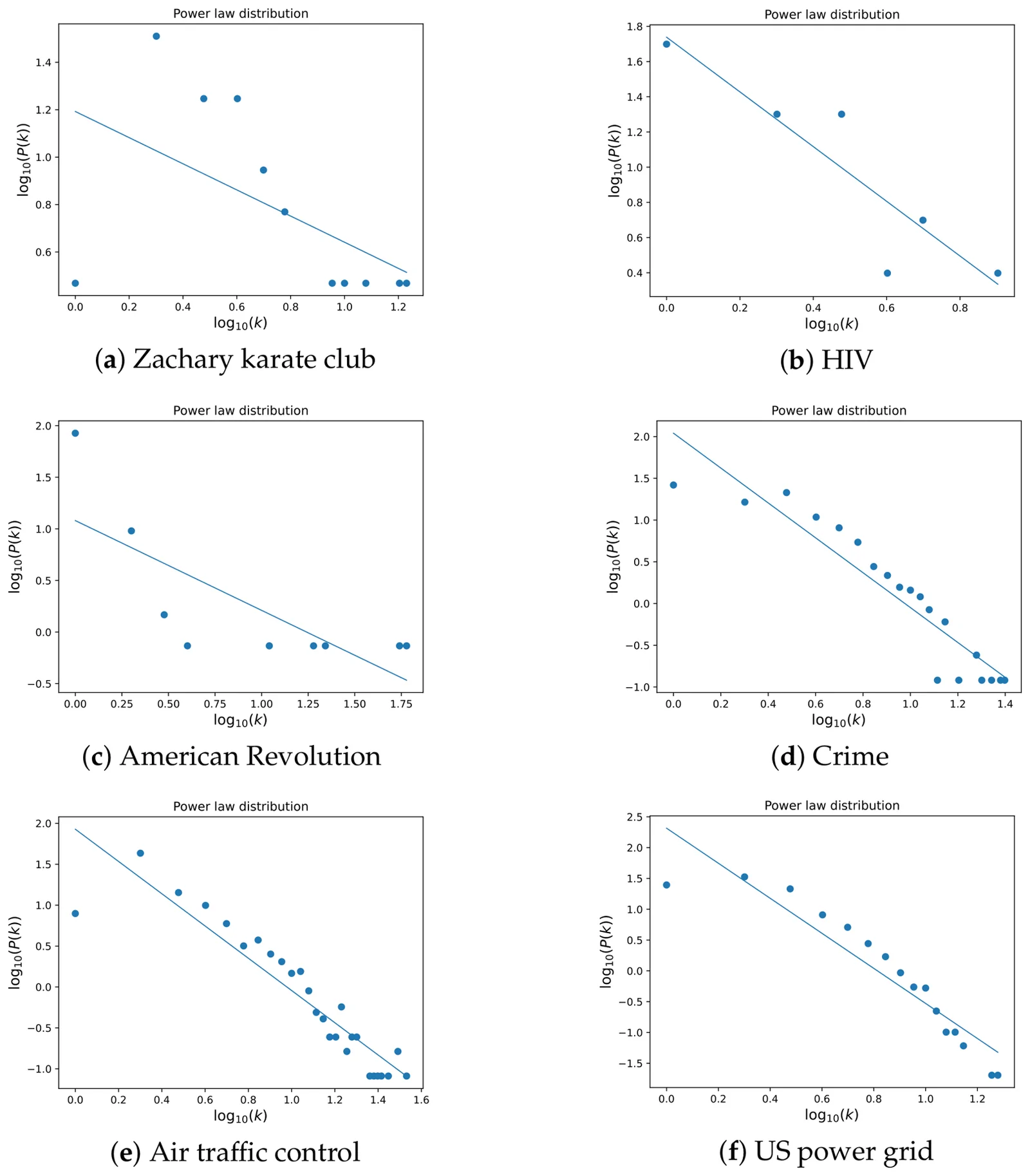
Power-law distribution plots of different real-world networks: (**a**) Zachary karate club, (**b**) HIV, (**c**) American Revolution, (**d**) Crime, (**e**) Air traffic control, and (**f**) US power grid. The horizontal axis represents the logarithm of node degree, i.e., $\log_{10}\left(k\right)$, while the vertical axis denotes the logarithm of the degree distribution, i.e., $\log_{10}\left(P\left(k\right)\right)$. Each point corresponds to the proportion of nodes with a given degree, and the fitted lines illustrate the underlying power-law behavior of the networks.

Figure 3 presents the degree distributions of the six real-world networks on a log–log scale to illustrate their overall distributional characteristics. For the three relatively small networks (Zachary karate club, HIV, and American Revolution), the limited number of nodes results in relatively few high-degree nodes, making the heavy-tailed characteristics less pronounced. Although a power-law-like tendency can be observed, the available data are insufficient to support a rigorous statistical claim of a power-law distribution. In contrast, the three larger networks (Crime, Air traffic control, and US Power Grid) exhibit more evident heavy-tailed degree distributions due to their larger network size and richer structural heterogeneity. Therefore, the log–log plots in this work are intended only to provide a qualitative illustration of the degree distribution characteristics rather than to establish that the experimental networks are strictly scale-free. Furthermore, the observed structural characteristics of the experimental networks, together with the computational efficiency considerations, the six-degrees-of-separation principle, and the sensitivity analysis presented in Section 3, collectively support the reasonableness of selecting a search step of 8 in this study.

Overall, these six types of networks exhibit significant differences in scale, connectivity, and local clustering characteristics, ranging from highly clustered small-scale social networks to low-density large-scale infrastructure networks. This structural diversity provides a comprehensive and robust experimental foundation for systematically evaluating the applicability and robustness of node importance identification methods across different types of networks.

Based on the dataset descriptions and network structure analysis above, it can be concluded that these datasets span a broad range of network sizes, densities, and structural characteristics, ranging from small and highly clustered social networks to large and sparse infrastructure networks. Such diversity provides a solid and reproducible experimental basis for the comprehensive evaluation of influential node identification methods.

### 4.2. Evaluation Metrics

Under the experimental design framework and evaluation setting, we employ three complementary metrics from different perspectives to comprehensively assess the performance of the proposed method and six baseline methods, including the time-dependent number of infected nodes F(t), the largest connected component ratio *G*, and Kendall’s correlation coefficient τ. These metrics respectively evaluate the variation in infected node dynamics during the spreading process, the structural robustness of the network under node removal, and the correlation and reliability among different indicators. A detailed comparative analysis is further conducted against the results of the six benchmark methods.

#### 4.2.1. The Susceptible–Infected (SI) Model

In the study of influential node identification in complex networks, the Susceptible–Infected (SI) model [45,53] is a classical spreading dynamics model used to characterize the propagation capability of nodes during information diffusion or disease transmission processes. In this model, all nodes are divided into two states, namely susceptible and infected, while the network size is assumed to remain constant. Let the number of susceptible nodes S(t) and infected nodes I(t) at time *t* be defined, respectively, where *N* denotes the total number of nodes in the network. The conservation relationship is given by the following:

(12) S(t)+I(t)=N,

During the spreading process, infected nodes transmit the infection to their susceptible neighbors with a transmission rate β, and the infected state is assumed to be irreversible. For completeness, the classical mean-field formulation of the SI model can be written as follows:

(13) $$\frac{dI(t)}{dt}=\beta I\left(t\right)\left(1-\frac{I(t)}{N}\right),$$

which describes the growth mechanism of the infected population over time. Although the above equation provides the theoretical background of the classical SI model, it is not used in our experiments. Instead, all spreading experiments are conducted using discrete SI simulations on the network topology. Within this simulation framework, node importance is evaluated according to its influence on the global diffusion process when acting as a seed node. For unified representation, the infected scale during the spreading process is defined as follows:

(14) F(t)=I(t),

where F(t) represents the diffusion capability of the seed node at time *t*. Furthermore, the importance of different nodes is quantified by comparing the time *t* required for the infection process to cover the entire network; a smaller *t* indicates that the corresponding node can more rapidly drive global diffusion, and therefore possesses stronger propagation capability and higher importance.

#### 4.2.2. The Maximum Connectivity Coefficient

In an undirected network, a connected subnetwork is defined as a subset of nodes in which any pair of nodes is connected by at least one path. The largest connected subnetwork, also known as the giant connected component, refers to the connected subnetwork containing the maximum number of nodes. To evaluate the rate of decline in network connectivity during the node removal process, the maximum connectivity coefficient *G* is commonly used, which is defined as the ratio between the number of nodes in the largest connected subnetwork and the total number of nodes in the network [46,54]. Let a network G=(V,E) contain *N* nodes, where $G_{\max}$ denotes the largest connected subnetwork, $|G_{\max}|$ denotes the number of nodes in $G_{\max}$, and |V| denotes the total number of nodes in the network. The maximum connectivity coefficient *G* is given by the following:

(15) $$G=\frac{\left|G_{\max}\right|}{\left|V\right|},$$

This coefficient *G* is used to quantify the proportion of nodes contained in the largest connected subnetwork relative to the entire network. When *G* approaches 1, it indicates that most nodes belong to a single connected component, implying strong network connectivity. Conversely, when *G* approaches 0, it suggests that the network is fragmented into multiple disconnected components, indicating weak connectivity. By sequentially removing nodes according to their rankings under different metrics and recording the variation of *G* at each step, the network disintegration process can be directly observed, i.e., the rate of decline in connectivity. In particular, if the removal of top-ranked nodes under a given metric leads to the fastest decrease in *G*, it indicates that the metric is more effective in identifying important nodes. This procedure provides an effective means of evaluating the accuracy and effectiveness of different node importance measures in identifying critical nodes, thereby offering valuable insights for network propagation analysis and critical node identification.

#### 4.2.3. The Kendall Correlation Coefficient

The Kendall correlation coefficient is a nonparametric statistical measure widely used to assess the consistency between two ranking sequences [47,55]. In this study, it is used to evaluate the correlation between the node importance rankings generated by structural-based methods and the spreading results of the SI model. Suppose the network contains *N* nodes, and two ranking sequences are given by $X=(x_{1},x_{2},\ldots ,x_{N})$ and $Y=(y_{1},y_{2},\ldots ,y_{N})$. Each node corresponds to a paired observation $\left(x_{i},y_{i}\right)$, forming a set of *N* paired elements. For any pair of paired elements $\left(\left(x_{i},y_{i}\right),\left(x_{j},y_{j}\right)\right)$, their relative order is examined. If the relative order is consistent in both sequences $x_{i}>x_{j}$ and $y_{i}>y_{j}$, or $x_{i}<x_{j}$ and $y_{i}<y_{j}$, the pair is defined as a *concordant pair*. If the relative order is opposite, i.e., $x_{i}>x_{j}$ and $y_{i}<y_{j}$, or $x_{i}<x_{j}$ and $y_{i}>y_{j}$, the pair is defined as a *discordant pair*. If $x_{i}=x_{j}$ or $y_{i}=y_{j}$, the pair is considered a tied pair. When multiple nodes obtain identical importance scores, they are treated as tied rather than being assigned an arbitrary ranking order. Accordingly, no additional tie-breaking strategy (e.g., node index or random ordering) is applied. During the correlation calculation, tied pairs are directly taken into account to ensure that the resulting Kendall correlation coefficient is not affected by arbitrary ordering of nodes with identical importance scores. The Kendall τ coefficient is then calculated as follows:

(16) $$\tau =\frac{2\left(n_{a}-n_{b}\right)}{N(N-1)},$$

where $n_{a}$ and $n_{b}$ denote the numbers of concordant and discordant pairs, respectively. The value of τ ranges from −1 to 1. When τ→1, it indicates a strong positive correlation between the two rankings, with high consistency. When τ→−1, it suggests that the rankings are nearly completely opposite. When τ→0, it implies weak or no correlation between the rankings. Since Kendall’s τ depends only on the relative order and not the absolute values of the rankings, it is particularly suitable for evaluating the consistency between structural ranking methods and epidemic spreading outcomes in complex networks.

### 4.3. Results

To validate the performance of the proposed CNC method, experiments are conducted using the network datasets introduced in Section 4.1. A systematic comparison with various classical centrality measures and related improved methods is carried out to evaluate the applicability and advantages of the proposed approach in complex networks from multiple perspectives. Specifically, in Section 4.3.1, information diffusion simulations based on the SI spreading model are performed to characterize the differences in node influence among various methods during the propagation process. In Section 4.3.2, a node removal experiment is designed to assess network robustness by analyzing the variation in the relative size of the largest connected component as critical nodes are progressively removed. In Section 4.3.3, the propagation results obtained from the SI model are compared with the ranking results of different methods, and the variation in Kendall’s correlation coefficient τ under different transmission probabilities is further investigated. The results of these multi-dimensional experiments demonstrate that the proposed CNC method achieves strong overall performance and exhibits significant advantages in identifying influential nodes in complex networks.

#### 4.3.1. Result 1: SI Model

Based on Section 4.2.1, the experimental setup is as follows. Seven different node importance identification methods are employed, and the top 10 ranked nodes selected by each method are used as initial infected seeds, while all remaining nodes are set as susceptible. The spreading process is implemented in discrete time steps, with the transmission probability set to β=0.03.

To evaluate the spreading performance of different methods, the infected scale F(t) is used as a dynamic metric to characterize the evolution of infected nodes over time. Each experiment is independently repeated 100 times, and the results are averaged to reduce stochastic fluctuations and improve stability. If the nodes selected by a given method can more efficiently accelerate full network coverage, the corresponding spreading time will be shorter, indicating stronger propagation capability and higher importance of the identified nodes. This experiment aims to systematically compare the effectiveness and robustness of different node identification methods under spreading dynamics.

**Figure 4:**
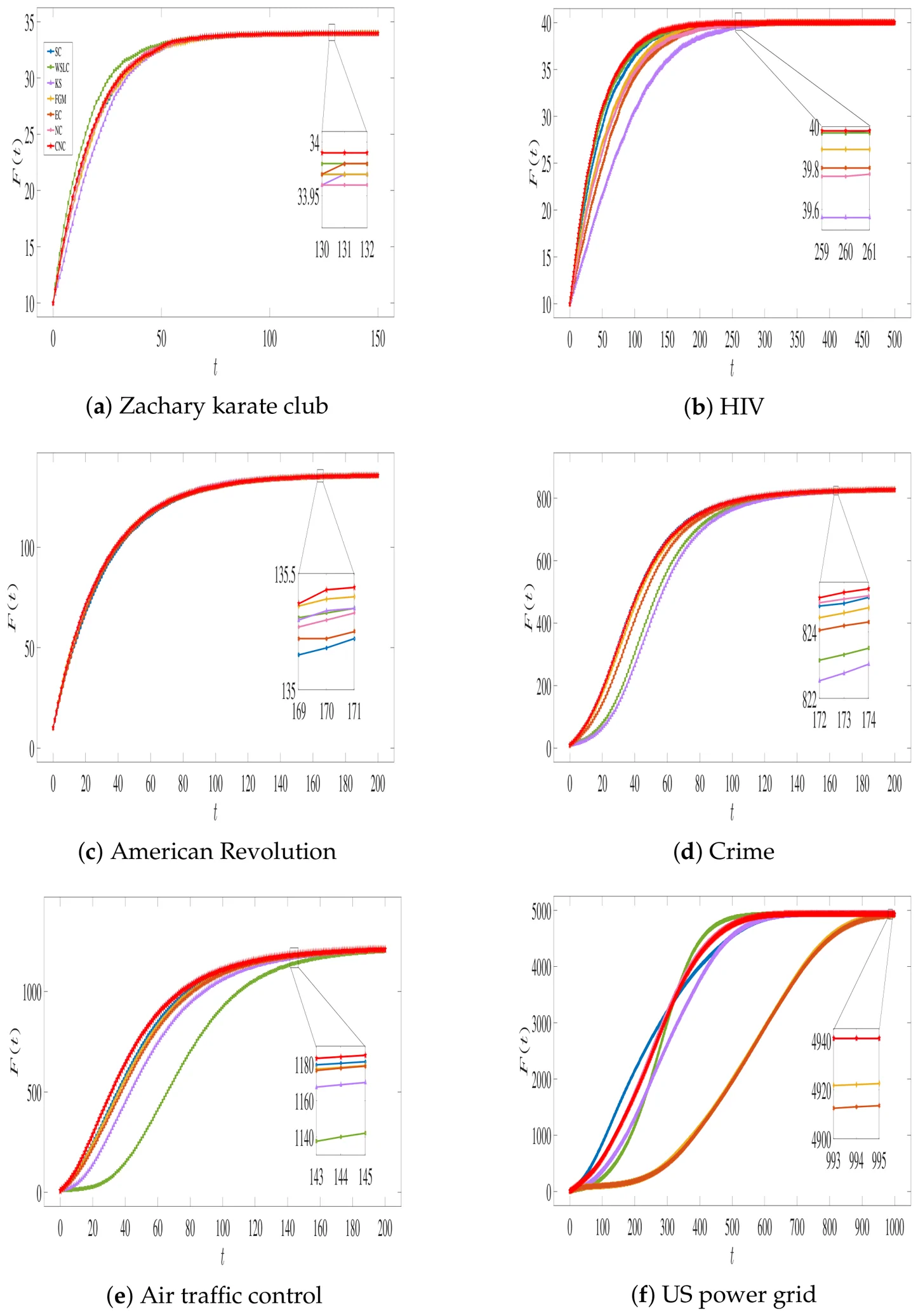
Comparison of the infection capability of the top 10 nodes selected by different methods across six real-world networks: (**a**) Zachary karate club, (**b**) HIV, (**c**) American Revolution, (**d**) Crime, (**e**) Air traffic control, and (**f**) US power grid.

Specifically, as shown in Figure 4a, in the Zachary karate club network, the propagation influence of the CNC method is second only to that of WSLC when t<50, and is approximately comparable to the infection strength of EC, while clearly outperforming the remaining methods. When t>50, CNC gradually becomes dominant in the spreading process and consistently outperforms the other six methods. Furthermore, it can be observed from the figure that, at t=130, the curve corresponding to CNC lies above all other methods, achieving full network infection first, which strongly demonstrates its superior capability in capturing propagation dynamics.

As shown in Figure 4b, in the HIV network, the CNC method consistently exhibits the best performance throughout the entire spreading process. In particular, when t<200, the propagation curve of CNC is significantly higher than those of the other six methods, demonstrating a clear and substantial advantage. When t>200, although the propagation abilities of the other methods gradually improve and approach that of CNC, the CNC curve remains consistently superior, with the gap narrowing but still clearly observable.

As shown in Figure 4c, in the American Revolution network, the overall spreading trends of different methods are relatively similar, while the CNC method consistently maintains a slight advantage. Specifically, when t<135, the infection scale of CNC is approximately comparable to those of NC and FGM, and is slightly higher than that of the remaining methods. When t>135, the advantage of CNC becomes increasingly evident and remains dominant in the subsequent process. From the subfigure, it can be observed that, at t=170, the curve corresponding to CNC reaches the highest position among all methods, achieving full network infection first, which further demonstrates its superior performance in evaluating spreading capability.

As shown in Figure 4d, in the Crime network, the CNC method exhibits progressively improving performance across different spreading stages. When t<100, its propagation ability is second only to SC and is comparable to that of NC, while clearly outperforming the remaining methods. When t>100, CNC gradually overtakes both SC and NC and maintains the best performance thereafter. From the subfigure, it can be observed that, at t=173, the curve corresponding to CNC lies above all other methods, further confirming its leading advantage in terms of propagation capability.

As shown in Figure 4e, in the Air traffic control network, the CNC method demonstrates strong competitiveness across different stages. When t<140, its propagation performance is approximately comparable to that of NC, while clearly outperforming the remaining methods. When t>140, CNC surpasses NC and consistently maintains the best performance throughout the subsequent spreading process. It is also observed that the KS method remains at the lowest level over the entire time horizon, with a substantial gap compared with other methods, indicating the weakest performance among all approaches.

As shown in Figure 4f, in the US power grid network, the CNC method exhibits progressively increasing advantages across different propagation stages. When t<800, its performance is slightly lower than that of SC and WSLC, and is comparable to NC, while still outperforming the other methods. When t>800, CNC gradually surpasses all competing methods and maintains a leading position thereafter. In addition, throughout the entire spreading process, both FGM and EC consistently perform poorly, with their corresponding curves remaining well below those of the other methods, indicating relatively weak propagation capability.

#### 4.3.2. Result 2: The Maximum Connectivity Coefficient

To further validate the effectiveness of different methods in influential node identification, we adopt the concepts the maximum connectivity coefficient. Under different centrality-based ranking strategies, a non-adaptive node removal strategy is adopted, where all nodes are first ranked in descending order according to their corresponding centrality scores. The obtained ranking remains fixed throughout the dismantling process, and nodes are sequentially removed following this predetermined order. The removal fraction is gradually increased, and the robustness of the network is characterized by the variation of the largest connected component ratio *G*. Specifically, *G* measures the extent of structural fragmentation during the node removal process; a faster decline in *G* indicates that the corresponding method is more effective in identifying structurally critical nodes. Based on the experimental results obtained from the six real-world networks, it can be observed that the value of *G* consistently decreases as the removal fraction increases, although the rates of decline differ significantly among different methods. Notably, during the early stages of node removal, when only a relatively small fraction of nodes has been removed, the proposed CNC method already exhibits a more rapid decrease in *G* than the competing methods. This indicates that the nodes identified by CNC play a more critical role in maintaining network connectivity. As the removal process continues, the superiority of the proposed method remains evident across all six networks, demonstrating its effectiveness in weakening network connectivity through the identification of structurally important nodes.

**Figure 5:**
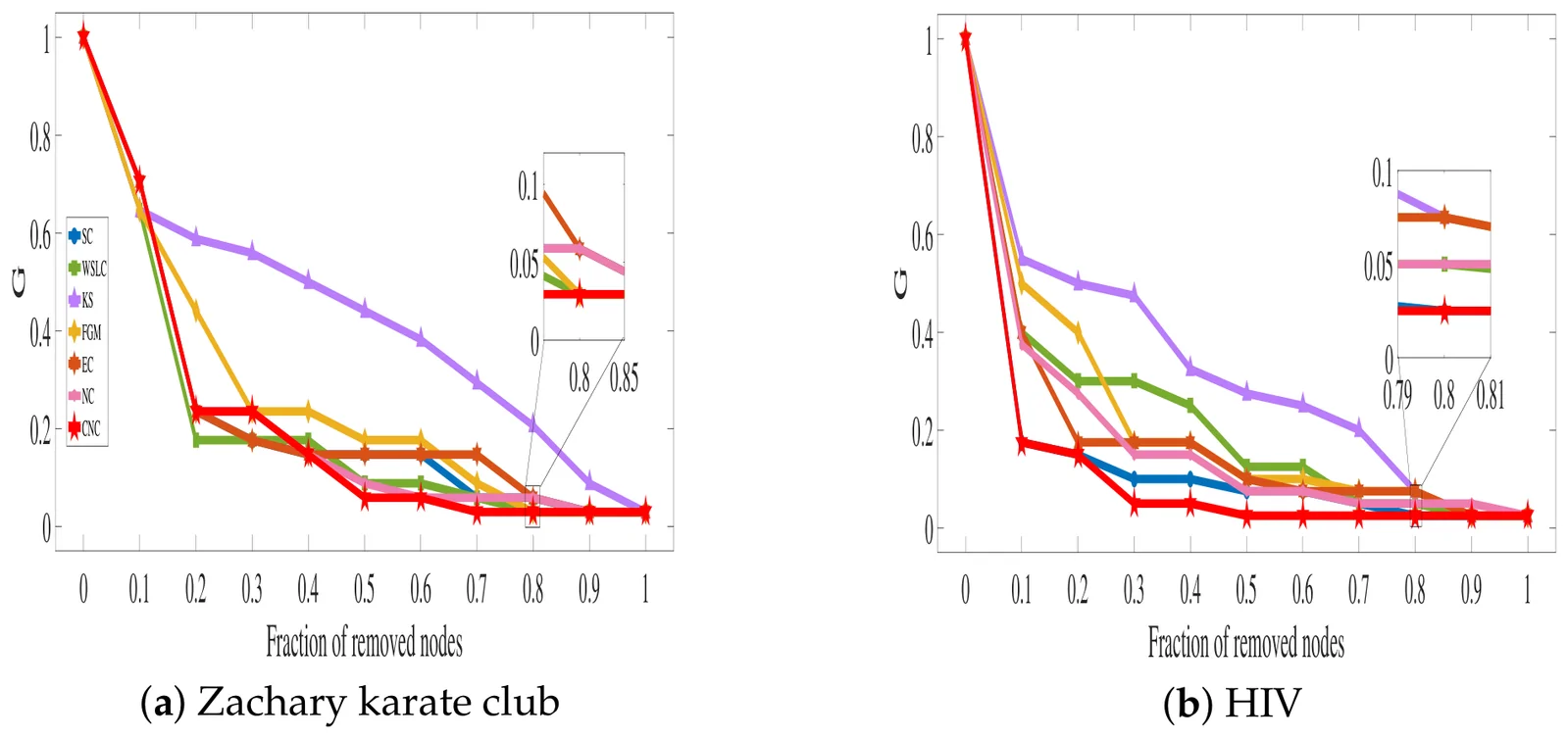

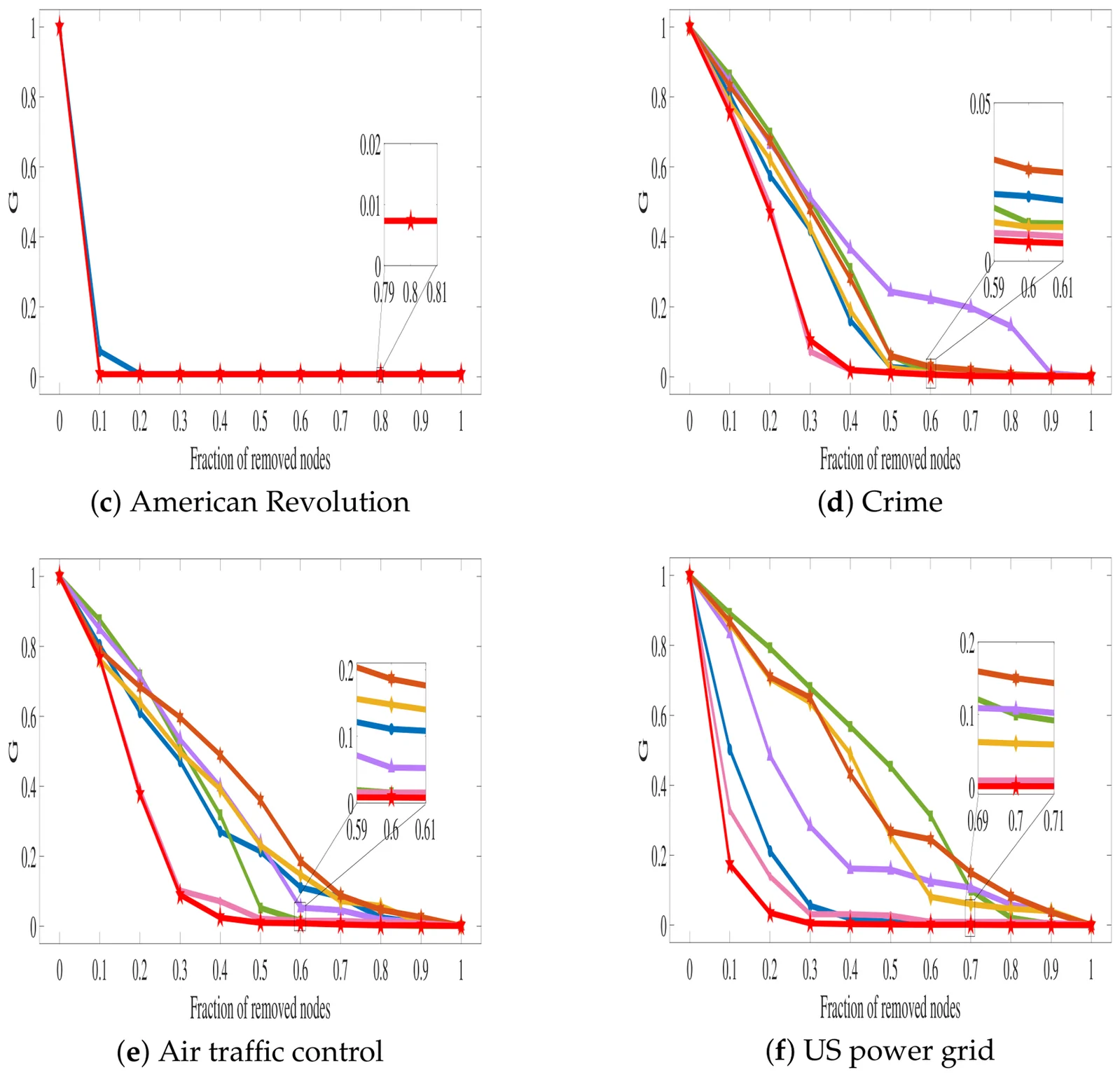
Comparison of the largest connected component ratio *G* as a function of the fraction of removed nodes across six real-world networks: (**a**) Zachary karate club, (**b**) HIV, (**c**) American Revolution, (**d**) Crime, (**e**) Air traffic control, and (**f**) US power grid.

Specifically, as shown in Figure 5a, in the Zachary karate club network, when the node removal fraction is less than 0.4, the WSLC and EC methods exhibit faster disruption in the early stage, as reflected by a more rapid decline in the largest connected component ratio *G*. When the removal fraction exceeds 0.4, the CNC method gradually becomes dominant and consistently outperforms the other methods in the subsequent process. Furthermore, it can be observed that, when the removal fraction reaches 0.8, the curve corresponding to CNC decreases to the lowest level and is the first to reduce the largest connected component ratio *G* to 0, demonstrating its significant advantage in network dismantling capability.

As shown in Figure 5b, in the HIV network, when the node removal fraction is less than 0.2, the CNC and SC methods exhibit similar disruption efficiency in the early stage, with nearly identical decline rates of the largest connected component ratio *G*, and both outperforming the other methods. When the removal fraction exceeds 0.2, the CNC method gradually demonstrates stronger dismantling capability and consistently maintains a leading position in the subsequent process. Furthermore, it can be observed that, when the removal fraction reaches 0.8, the curve corresponding to CNC drops to the lowest level and is the first to reduce the largest connected component ratio *G* to 0, further highlighting its significant advantage in network dismantling. In addition, the KS method shows the slowest decline throughout the process, indicating relatively weak capability in disrupting network structure.

As shown in Figure 5c, in the American Revolution network, when the node removal fraction is less than 0.1, the curve corresponding to CNC rapidly drops to the lowest level and is the first to reduce the largest connected component ratio *G* to 0. In this stage, the WSLC, KS, FGM, EC, and NC methods exhibit performance consistent with CNC, with their curves almost completely overlapping. In contrast, the SC method shows a slower decline and fails to reduce *G* to 0 within this range; it only achieves complete fragmentation when the node removal fraction reaches 0.2.

As shown in Figure 5d, in the Crime network, when the node removal fraction is less than 0.4, the curve corresponding to CNC rapidly drops to the lowest level and is the first to reduce the largest connected component ratio *G* to 0. In this stage, the NC method exhibits performance comparable to that of CNC, with their curves nearly overlapping. When the removal fraction reaches 0.6, the *G* values of WSLC, SC, FGM, and EC gradually decrease to near zero. In contrast, the KS method shows the slowest decline, not reaching the lowest level until the removal fraction increases to 0.9.

As shown in Figure 5e,f, in the Air traffic control and US power grid networks, the curve corresponding to CNC consistently remains the lowest throughout the entire node removal process and is the first to reduce the largest connected component ratio *G* to 0. The NC method exhibits performance largely comparable to that of CNC, while the remaining five methods show relatively slower decline rates, indicating weaker network dismantling capability.

#### 4.3.3. Result 3: Kendall Correlation Coefficient

**Figure 6:**
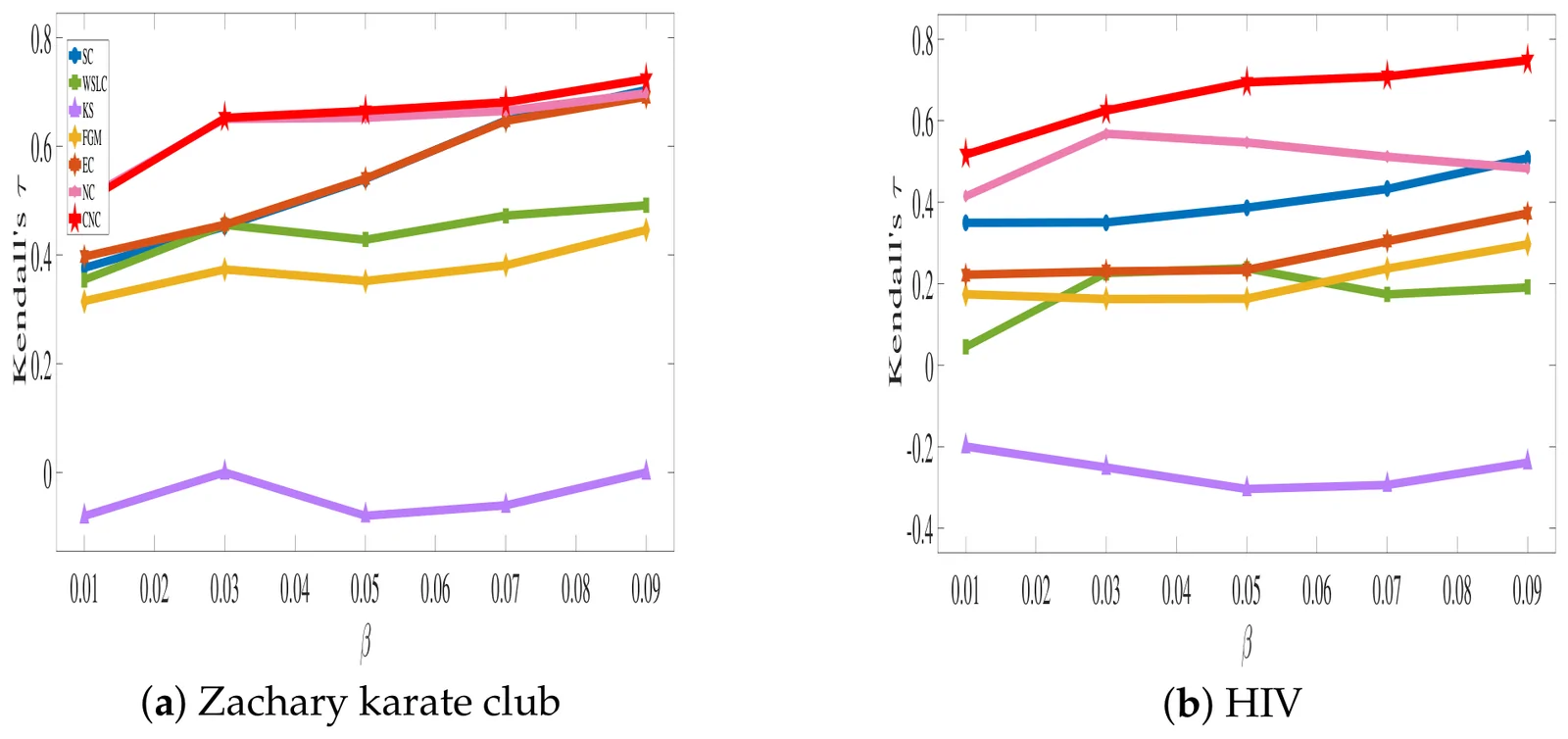

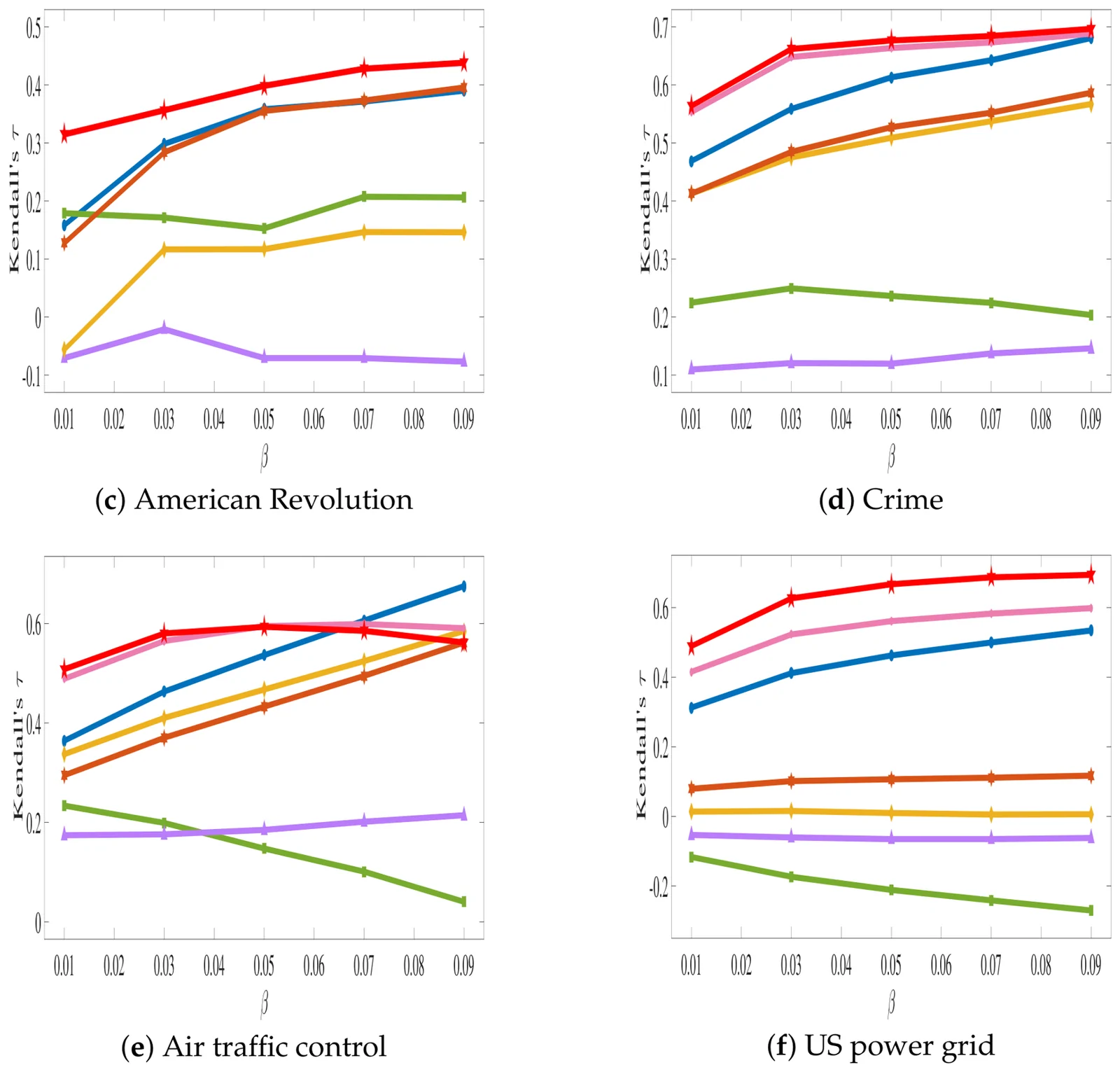
Kendall’s correlation coefficient τ of different methods as a function of the infection probability β across six real-world networks: (**a**) Zachary karate club, (**b**) HIV, (**c**) American Revolution, (**d**) Crime, (**e**) Air traffic control, and (**f**) US power grid.

To improve the reliability and objectivity of the experimental results, each node in the network is individually selected as the initial infected seed to evaluate its spreading capability under the SI model. For each seed node, the SI spreading process is independently simulated 100 times, and the average spreading influence is adopted as the reference influence score of that node. Based on these average spreading influences, the ground-truth node ranking is constructed for each network. To comprehensively evaluate the consistency between the proposed method and the actual spreading dynamics, multiple simulation scenarios are generated by systematically varying the infection probability β. For each value of β, every simulation is independently repeated 100 times to reduce the influence of stochastic fluctuations, and the averaged results are used for subsequent analysis. The Kendall correlation coefficient between the ranking produced by each centrality measure and the ground-truth spreading ranking is then calculated. The overall averaged results are presented in Figure 6. In general, a higher value of τ indicates stronger consistency between the node ranking generated by a given method and the actual spreading influence measured by the SI simulations, thereby demonstrating better effectiveness in identifying influential nodes.

As shown in Figure 6a, in the Zachary karate club network, the Kendall correlation coefficient τ varies significantly across different centrality methods as the infection probability β changes. Specifically, when 0.01<β<0.09, the CNC method achieves the highest consistency among all compared approaches. Within this range, the CNC curve is nearly identical to that of the NC method and is consistently higher than those of the other five methods, indicating that both CNC and NC can more accurately capture node spreading importance in this network. In contrast, the KS method exhibits the weakest performance across the entire range of β, with its τ value remaining the lowest throughout.

As shown in Figure 6b, in the HIV network, the CNC method consistently achieves significantly higher Kendall correlation coefficients than the other six methods across the entire range of β. The NC method ranks second and remains close to CNC, whereas the KS method performs the worst and even exhibits a clear negative correlation. These results indicate that the ranking produced by CNC is more consistent with the true spreading influence captured by the SI model, demonstrating a higher accuracy in characterizing node importance during the propagation process. In contrast, the KS method shows limited discriminative ability for node influence, leading to a substantial deviation from the actual spreading dynamics.

As shown in Figure 6c, in the American Revolution network, the CNC method consistently achieves the best performance across the entire range of β, with its Kendall correlation coefficient curve remaining higher than those of all other methods. The SC and EC methods exhibit similar performance, although both are inferior to CNC. In contrast, the KS method performs the worst, with its curve remaining the lowest among all methods.

As shown in Figure 6d, in the Crime network, the CNC method consistently achieves the best performance across the entire range of β. The NC method exhibits a similar trend to CNC, although its values are slightly lower overall. As β increases, the SC curve gradually approaches those of CNC and NC, but still remains below CNC. In contrast, the KS method continues to perform the worst; however, no negative correlation is observed in this network.

As shown in Figure 6e, in the Air traffic control network, when 0.01<β<0.05, the CNC method achieves the best performance, and the NC method follows a similar trend but remains slightly lower than CNC. As β increases to the range of 0.05<β<0.09, the curves of the NC and SC methods gradually surpass that of CNC; however, the overall differences among these methods remain relatively small.

As shown in Figure 6f, in the US power grid network, when 0.01<β<0.09, the CNC method achieves the best performance, significantly outperforming the other methods. Meanwhile, the curves corresponding to different methods do not intersect within this range, indicating clear and pronounced differences among the compared metrics.

Overall, the CNC method consistently demonstrates stable and superior performance across the six real-world networks, maintaining leading or near-leading results under different infection probabilities and network structures. Meanwhile, the NC method yields results highly consistent with those of CNC, indicating strong agreement and comparability in characterizing node spreading influence. In contrast, the KS method performs the worst across all networks, showing low consistency with spreading dynamics-based rankings. This discrepancy is largely attributed to differences in how these methods utilize network structural information. In particular, the simplified formulation of the KS method limits its ability to distinguish fine-grained structural variations, thereby leading to relatively weaker overall performance.

## 5. Conclusions and Discussion

This paper proposes a hybrid centrality measure that integrates information from both smallest-cycle structures and non-smallest-cycle structures associated with each target node. This study addresses the problem of identifying influential nodes in complex networks by proposing a hybrid centrality measure that integrates information from both smallest-cycle structures and non-smallest-cycle structures associated with each target node (CNC). From a multi-scale structural perspective, the proposed method jointly models the smallest cycle structures in which a node participates and the non-cycle information within its neighborhood, thereby providing a more comprehensive characterization of a node’s structural role and functional significance in complex networks. Specifically, the Number of Smallest Cycle is employed to quantify the extent of a node’s embedding within locally closed structures, while the number of neighboring nodes not involved in smallest cycles is used to capture structural heterogeneity and the node’s capability for extended connectivity. These two complementary aspects are then integrated through a weighted fusion scheme to construct a unified node importance metric. Compared with conventional approaches that rely on a single structural feature, CNC effectively combines information from both local closure and openness, thereby improving the completeness and accuracy of node importance characterization.

To evaluate the effectiveness of the proposed method, extensive experiments were conducted on six real-world complex networks, and comparative analyses were performed against several classical and improved centrality measures. From the perspective of spreading dynamics, under the SI (Susceptible–Infected) model, nodes identified by the CNC method were found to facilitate more efficient information diffusion, leading to faster growth and wider coverage of the infected population. This indicates that CNC can accurately identify nodes that play a critical role in the propagation process. From the perspective of network structural robustness, in targeted attack experiments based on progressive node removal, the largest connected component ratio *G* decreased more rapidly when nodes were removed according to the CNC ranking, demonstrating that the method effectively prioritizes nodes with a decisive impact on overall network connectivity. In terms of ranking consistency, results based on the Kendall correlation coefficient τ show that CNC exhibits higher agreement with SI-based spreading dynamics, further confirming its ability to better reflect the true spreading influence of nodes. Overall, these results demonstrate that the proposed method achieves superior performance not only in ranking accuracy, but also in structural disruption analysis and dynamical consistency, while maintaining strong robustness and stability.

Beyond the benchmark evaluations, the proposed method also has potential applicability in a variety of real-world network analysis tasks. For example, in epidemic prevention and control, accurately identifying influential spreaders can facilitate targeted intervention strategies and effectively suppress disease transmission, as demonstrated in recent studies on COVID-19 propagation. In criminal intelligence analysis, identifying structurally critical individuals can improve the efficiency of dismantling resilient criminal organizations while minimizing intervention costs. Furthermore, in large-scale real-world networks, effective influential node identification provides essential support for applications such as network monitoring, resource allocation, and information dissemination. Therefore, the superior performance of the proposed CNC method in spreading dynamics, structural robustness, and ranking consistency not only demonstrates its effectiveness from a methodological perspective, but also suggests its potential value for practical applications involving network control, intervention, and optimization.

Overall, compared with traditional centrality measures such as SC, WSLC, KS, FGM, EC, and NC, the key advantage of CNC lies in its ability to simultaneously characterize the constraining effects of cycle structures and the expansive properties of non-cycle neighborhoods, thereby enabling a unified representation of multi-scale structural information. Although several baseline methods may exhibit comparable or slightly better performance at the early stage of node removal, their effectiveness degrades rapidly as the level of perturbation increases, whereas CNC consistently maintains stable and superior performance. These findings suggest that integrating smallest-cycle and non-smallest-cycle features is beneficial for identifying critical nodes that preserve network functionality under varying disruption conditions, offering a more comprehensive and effective framework for influential node identification in complex networks. Integrating smallest-cycle and non-smallest-cycle features provides a more comprehensive characterization of a node’s role in complex networks.

## Figures and Tables

**Table 1:** The real-world networks used for experiments.

Network	*N*	*E*	〈k〉	〈d〉	*C*	ρ	*L*
Zachary karate club	34	78	4.588	2.408	0.571	0.139	5
HIV	40	41	2.050	4.473	0.042	0.053	10
American Revolution	136	157	2.309	2.688	0.093	0.017	5
Crime	829	1473	3.554	5.040	0.006	0.004	10
Air traffic control	1226	2408	3.928	5.929	0.068	0.003	17
US power grid	4941	6594	2.669	18.989	0.080	0.001	46

## Data Availability

The original contributions presented in this study are included in the article. Further inquiries can be directed to the corresponding author.
